# Toxicological Stability of *Piper aduncum* L. Essential Oil During Storage Against Stored-Product Insect Pests

**DOI:** 10.3390/insects17101084

**Published:** 2026-10-20

**Authors:** Gabriela da Silva Tamwing, Guilherme Calado de Almeida, Josiane Moura do Nascimento, Roger Ventura Oliveira, Cleyton Silva de Araújo, Giovana Lopes da Silva, Mariano Oscar Aníbal Ibañez Rojas, Francisco José Teixeira Gonçalves, Leonardo Baltazar Cantanhede, Douglas Rafael e Silva Barbosa, Marcela Silva Carvalho, Gutierres Nelson Silva, Lucas Martins Lopes, Maria Regiane Araujo Soares, Adalberto Hipólito de Sousa

**Affiliations:** 1 Center for Biological and Natural Sciences, Universidade Federal do Acre, Rio Branco 69920-900, AC, Brazil; gabrielat.agro@gmail.com (G.d.S.T.); cleytonsdearaujo@gmail.com (C.S.d.A.);; 2 Instituto Federal do Maranhão, Campus Codó, Codó 65400-000, MA, Brazil; 3 Instituto Federal de Mato Grosso do Sul, Campus Naviraí, Naviraí 79950-000, MS, Brazil; 4 Instituto Federal de Mato Grosso do Sul, Campus Nova Andradina, Nova Andradina 79750-000, MS, Brazil; gutierres.silva@ifms.edu.br; 5 Universidade Federal do Piauí, Campus Amílcar Ferreira Sobral, Floriano 64800-000, PI, Brazil; regiane@ufpi.edu.br; 6 Department of Plant Science, Agricultural Science Center, Universidade Federal do Piauí, Teresina 64049-550, PI, Brazil

**Keywords:** bioinsecticide, dillapiole, dose–response, residual toxicity, light exposure, temperature, shelf life

## Abstract

*Piper aduncum* L. essential oil (PAEO) has insecticidal potential, but its performance under different storage conditions remains incompletely characterized. We compared PAEO stored at 5, 20, or 35 °C protected from light and, in a separate experiment at 20 °C, under light-protected or light-exposed conditions. Adults of four stored-product beetle species were tested after 4, 8, and 12 months. Initial GC-MS characterization showed that dillapiole accounted for 70.34% of the chromatographic area. Replicate-level mortality counts were analyzed using binomial-probit models. Assessment time was associated with concentration–mortality responses for all four species; the trajectories were species dependent and often non-monotonic. Exploratory within-assessment model comparisons generally showed higher median lethal concentrations for the bottles stored at 35 °C or exposed to light at 4 and 8 months, although these patterns differed by species. The storage experiments were not factorial. Each condition had one oil bottle, and differences between assessment dates cannot be attributed solely to oil aging because concurrent fresh-oil controls were unavailable. PAEO remained insecticidally active at each assessment, but chemical changes and their mechanisms were not tested longitudinally.

## Simple Summary

Plant-derived insecticides could provide alternatives for controlling beetles that damage stored grains, but their performance during storage must be established. We tested essential oil from *Piper aduncum* leaves against four stored-product beetles after 4, 8, and 12 months under different temperatures or light conditions in individual storage bottles. The oil caused insect mortality at all assessment dates, and responses varied among insect species and treatments. The bottles kept at higher temperature or exposed to light generally showed lower activity in several comparisons during the first eight months. However, the experiments evaluated temperature and light separately, and the observed differences among assessment dates may also reflect changes in the insect populations tested. Chemical composition was measured only before storage. These findings support further evaluation of the oil as a botanical insecticide, although the exploratory comparisons do not demonstrate generalizable storage-treatment effects or performance under commercial storage conditions.

## 1. Introduction

Essential oils are complex mixtures of volatile, lipophilic secondary metabolites produced by plants [1,2,3]. Their chemical diversity underlies broad applications in medicine, perfumery, cosmetics, and food-related products [1,2]. Storage temperature can also affect their composition and quality [4]. Essential oils exhibit a range of biological activities, including antifungal [5], antibacterial [6], antiparasitic [7], and insecticidal effects [8].

The insecticidal activity of *Piper aduncum* L. essential oil (PAEO) has been investigated for more than two decades as a botanical alternative for managing insect pests, including species associated with stored grains and other stored products [9,10,11,12,13]. Its biological effects are closely related to a composition rich in phenylpropanoids and terpenoids [14], although the magnitude and persistence of activity may depend on both the initial chemical profile and subsequent storage conditions.

Although partly genetically determined, essential-oil yield and composition can vary with geographic origin, edaphoclimatic conditions, harvest season, phenological stage [3,15,16], and postharvest procedures such as drying and extraction [17,18,19]. After extraction, additional changes may occur during storage because many constituents are volatile and susceptible to oxidation or other degradation processes.

Storage duration and environmental conditions can therefore affect both the physicochemical properties and the biological activity of essential oils [20,21]. Packaging, temperature, humidity, light, and oxygen availability are among the main factors that can alter essential-oil quality during storage [22,23,24,25], with possible consequences for toxicity to arthropods [26]. Because storage conditions vary among producers, laboratories, and end users, defining conditions that preserve essential-oil quality and biological activity is important for practical shelf-life assessment [20,27].

Information on the storage behavior of PAEO remains comparatively limited. General literature on essential-oil stability and safety recognizes storage and handling as important determinants of oxidation and shelf life [20,23,28]. In Amazonian *Piper* oils, Negreiros et al. [29] reported variation in the major constituent during storage, with dillapiole reaching its highest relative abundance after nine months in transparent bottles exposed to environmental conditions. Fazolin et al. [30] evaluated PAEO for four years and reported relatively stable dillapiole content together with maintained toxicity against *Spodoptera frugiperda*. However, evidence remains limited for the first year of storage under controlled temperature and light conditions when PAEO is used against multiple stored-product insect species. This distinction is practically relevant because target species differ markedly in susceptibility and because storage conditions may affect residual bioactivity before application.

This study compared concentration–mortality responses of PAEO assessed after 4, 8, and 12 months under three light-protected storage temperatures or, separately, two light-exposure conditions at 20 °C against *Sitophilus zeamais* (Coleoptera: Curculionidae), *Tribolium castaneum* (Coleoptera: Tenebrionidae), *Oryzaephilus surinamensis* (Coleoptera: Silvanidae), and *Cryptolestes ferrugineus* (Coleoptera: Cucujidae). The initial oil profile was characterized by GC-MS. Changes among assay dates are interpreted as observed toxicological variation rather than an unconfounded causal effect of storage duration.

## 2. Materials and Methods

### 2.1. Insect Rearing

*Sitophilus zeamais*, *T. castaneum*, *O. surinamensis*, and *C. ferrugineus* were reared from specimens collected in storage units located in Rio Branco, Acre, Brazil. The insects were maintained in 1.5 L glass bottles sealed with perforated plastic lids lined with organza fabric. Colonies were maintained at 25 ± 2 °C and 70 ± 5% relative humidity under a 24 h scotophase. The nominal temperature for insect rearing and subsequent bioassays was therefore 25 °C, although the reported operating tolerances differed.

Corn grains with a moisture content of 13% (wet basis) were used as the food substrate. Before use, the grains were treated with phosphine (PH_3_) and stored under refrigeration to prevent reinfestation. *Sitophilus zeamais* was reared on whole grains, whereas *T. castaneum*, *O. surinamensis*, and *C. ferrugineus* were maintained on ground grains.

### 2.2. Plant Material and PAEO Extraction

PAEO extraction was carried out at the Laboratory of Natural Products of the Technology Foundation of the State of Acre (Fundação de Tecnologia do Estado do Acre, FUNTAC), whereas the toxicological bioassays were conducted at the Laboratory of Integrated Pest Management, Federal University of Acre (Universidade Federal do Acre, UFAC), Rio Branco campus.

Plant material was collected between 06:00 and 07:00 h from adult *P. aduncum* specimens approximately five years old at the UFAC campus in Rio Branco, Acre, Brazil (9°57′34.9″ S, 67°51′30.6″ W). A voucher specimen was deposited in the UFACPZ Herbarium under registration number UFACPZ 20,646. Species identification was confirmed by Elsie Franklin Guimarães of the Rio de Janeiro Botanical Garden Herbarium (Herbarium RB). Plants were harvested 0.4 m above ground level. Leaves were separated, dried in an oven at 45 °C until constant moisture content, manually fragmented, and stored in 10 kg plastic bags.

The essential oil was obtained by hydrodistillation in a Clevenger apparatus connected to a refrigeration system and a 5 L round-bottom flask placed on a ceramic heating mantle. For each extraction, 150 g of dried leaf material was mixed with distilled water. The mixture was brought to a boil, and distillation continued for approximately 240 min. PAEO and hydrolate were collected in Erlenmeyer flasks. Related *P. aduncum* leaf-processing and Clevenger extraction procedures have been described previously [13].

The essential oil was separated by decantation in a separatory funnel and dried with anhydrous sodium sulfate (99%, Synth, Diadema, SP, Brazil). Samples were then transferred to amber bottles and stored under the predefined experimental conditions.

### 2.3. Chemical Characterization of PAEO

Gas chromatography coupled with mass spectrometry (GC-MS) was performed at the Department of Technical-Scientific Police of the Institute of Forensic Analysis (Instituto de Análise Forense, IAF), Civil Police of Acre.

Analyses were conducted using an Agilent 7890A gas chromatograph coupled to a 5975C mass selective detector (Agilent Technologies, Santa Clara, CA, USA). PAEO was diluted to 2% in methanol and injected in split mode into an HP-5MS capillary column (30 m × 0.25 mm internal diameter × 0.25 µm film thickness) using helium as the carrier gas. The injector temperature was 290 °C. The oven program started at 80 °C (5 min), increased at 4 °C min^−1^ to 285 °C, and was held at 285 °C for 40 min. The detector and interface were maintained at 290 °C. Electron-impact ionization was operated at 70 eV, and mass spectra were acquired from 30 to 600 Da.

Constituents were identified by comparing mass spectra with the instrument library, the National Institute of Standards and Technology (NIST) database, and published essential-oil literature [31]. Linear retention indices (LRI) relative to an n-alkane series (C_10_–C_40_, Fluka Analytical, Buchs, Switzerland) and Kovats indices (KI) were calculated using established reference data [31]. The oil was characterized by a single GC-MS analysis immediately after extraction; no further chromatographic analyses were performed at 4, 8, or 12 months.

### 2.4. Storage Conditions and Toxicological Stability Bioassays

**Figure 1:**
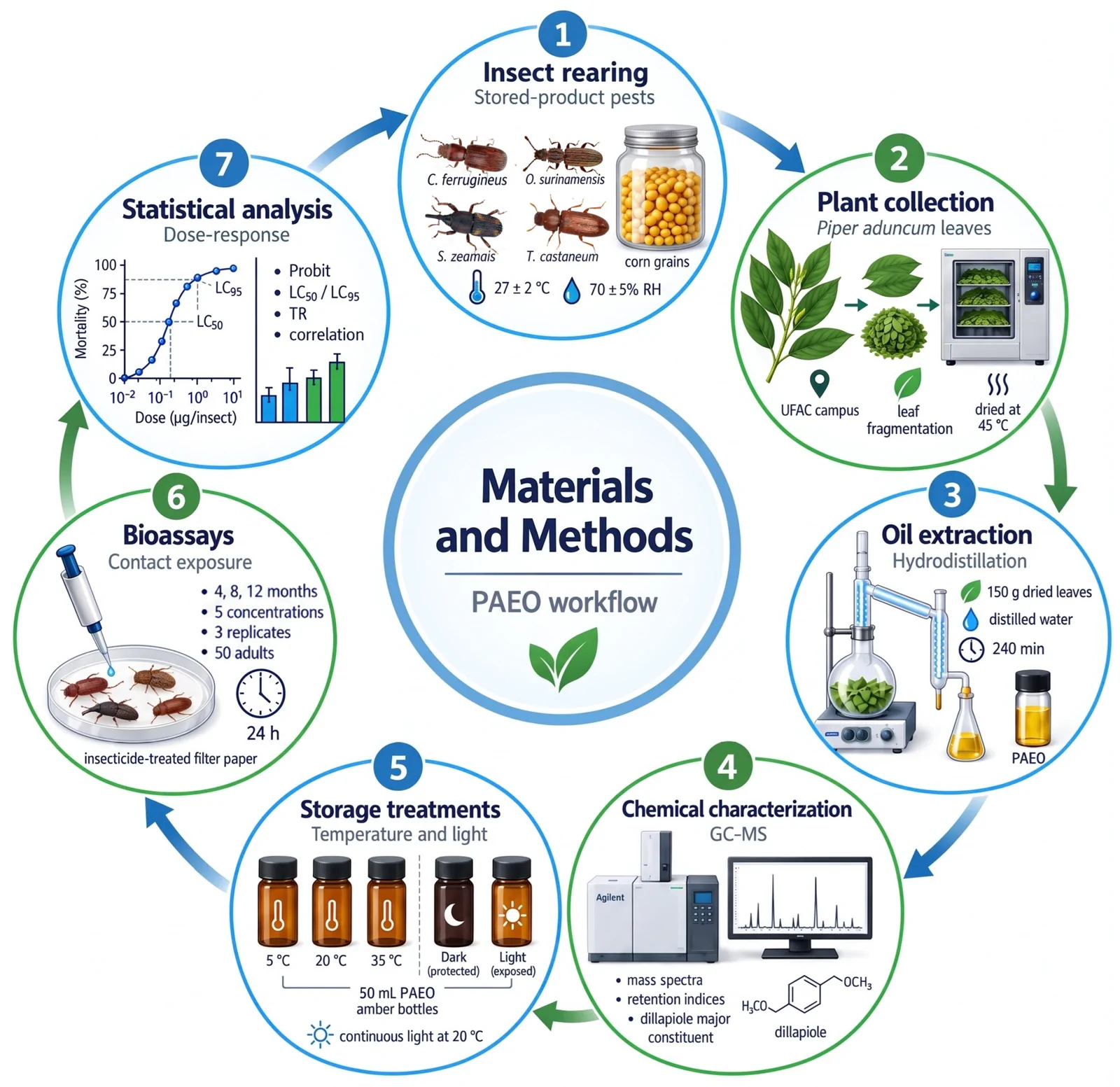
Experimental workflow used to evaluate the toxicological stability of *Piper aduncum* L. essential oil (PAEO) during storage against stored-product insect pests. The workflow summarizes insect rearing, plant collection and leaf preparation, PAEO extraction by hydrodistillation, initial chemical characterization by GC-MS, temperature- and light-based storage conditions, concentration-mortality bioassays conducted after 4, 8, and 12 months, and statistical analysis.

The overall experimental workflow is summarized in Figure 1. For the temperature experiment, one sealed amber glass bottle containing 50 mL of PAEO was allocated to each storage temperature and wrapped in aluminum foil to exclude light. Bottles were stored at 5.0 ± 1 °C under refrigeration, 20 ± 2 °C in a climatic chamber, or 35 ± 2 °C in a climatic chamber. The 5 °C treatment represented conventional refrigerated storage; the 20 and 35 °C conditions represented moderate and elevated indoor storage temperatures. The selected temperatures follow previous essential-oil storage work, including Moura et al. [21] and related stability studies [4,27]. All three temperature treatments were protected from light; this experiment did not test the effect of temperature under illumination.

For the separate light-exposure experiment, one 50 mL PAEO bottle per light condition was kept at 20 ± 2 °C in a BOD-type climatic chamber (model 347 CD, Fanem, São Paulo, Brazil). The illuminated treatment used continuous light from 100 W lamps (6500 K; nominal luminous flux 1521 lm; Philips, São Paulo, SP, Brazil), with bottles positioned 70 cm from the light source. Light-protected PAEO was stored in amber bottles wrapped in aluminum foil, whereas light-exposed PAEO was stored in unwrapped clear glass bottles, following a procedure adapted from [21]. The light experiment was held at 20 °C to compare protected and illuminated storage under the same temperature, rather than to assess a range of light intensities. Temperature and light were therefore analyzed as distinct experiments, not a fully crossed 3 × 2 factorial; additive or interaction effects between these factors cannot be inferred. The two light treatments also differed in container transparency and covering. Each bottle was opened every four months to withdraw the oil needed for bioassays, then closed and returned to its assigned storage condition. No closed sampling system was used.

Concentration–mortality bioassays were conducted after 4, 8, and 12 months of storage at 25 ± 5 °C and 70 ± 5% relative humidity, with the same nominal temperature used for insect rearing (Section 2.1). Preliminary tests identified ranges spanning partial mortality, and six concentrations were then evaluated for each species and assessment date. Across the complete dataset, concentrations ranged from 0.079 to 2.889 µL cm^−2^ for *S. zeamais*, 0.005 to 0.713 µL cm^−2^ for *O. surinamensis*, 0.002 to 0.013 µL cm^−2^ for *C. ferrugineus*, and 0.458 to 5.032 µL cm^−2^ for *T. castaneum*. The bioassay procedure followed the general contact-exposure approach previously used for PAEO against stored-product beetles [13].

Each bioassay experimental unit was a Petri dish (9 cm diameter × 1.5 cm height) lined with filter paper, treated with 1000 µL of the appropriate PAEO solution in acetone. For each species, storage condition, and assessment time, three control Petri dishes received acetone alone. Control mortality was 0% in every assessment. Following solvent application, dishes were left open for approximately 5 min to allow evaporation, as established in preliminary tests. Three independent Petri-dish replicates containing 50 adults each were available per concentration at 4 and 8 months for all species. At 12 months, three replicates per concentration were available for *C. ferrugineus*, *O. surinamensis*, and *S. zeamais*, whereas two were used for *T. castaneum* because fewer insects were available from the laboratory colony at this assessment. These replicates were independent Petri dishes within each concentration, whereas each storage condition was represented by one oil bottle. As control mortality was zero, no correction of the observed mortality counts was required.

Fifty unsexed adults (1–48 h old) were introduced into each dish, which was immediately covered. Mortality was recorded after 24 h. Insects were considered dead when they failed to respond to gentle stimulation with a brush or were unable to move.

### 2.5. Statistical Analysis

Statistical analyses were conducted in Python 3.13 within the Spyder 6 integrated development environment (IDE), using statsmodels 0.14.6 and SciPy 1.17.0. Analyses used the original numbers of dead and surviving insects in each Petri dish, not percentages averaged across replicates. Recorded control mortality was zero; accordingly, no Abbott correction was applied.

For each combination of species, storage condition, and assay date, concentration–mortality data were fitted using a binomial generalized linear model with a probit link. PAEO concentration (µL cm^−2^) was ${log}_{10}$-transformed, resulting in the model:

(1) $$probit\left(p\right)=\alpha +\beta \log_{10}\left(C\right),$$

where p is the probability of mortality, C is the PAEO concentration (µL cm^−2^), α is the intercept, and β is the slope of the concentration–mortality relationship.

Model adequacy was evaluated using Pearson residual dispersion and the Pearson goodness-of-fit test. When extra-binomial variation was detected, the covariance matrix used to estimate standard errors and confidence intervals for lethal concentrations was scaled by max(1, Pearson dispersion). Models showing extra-binomial variation and/or significant lack of fit are identified in the corresponding tables.

Temperature and light were analyzed as separate storage experiments because the design was not a fully crossed 3 × 2 factorial. Within each species, responses measured after 4, 8, and 12 months were analyzed using binomial-probit models with log_10_-concentration, categorical assay time, storage condition, and their interactions. The full model included log_10_-concentration × assay time × storage condition. Hierarchical comparisons with appropriate reduced models were performed using dispersion-adjusted F tests based on deviance differences. These tests characterize differences among the assayed insect responses conditional on the particular stored-oil bottles, rather than independently replicated storage-treatment effects. Assay time is an indicator of the assessment occasion: its estimated effect is potentially confounded with insect cohort, rearing batch, and other date-specific conditions, and is not interpreted as a pure causal effect of PAEO aging.

Exploratory storage-condition contrasts within each assessment date were additionally examined using likelihood-ratio comparisons of nested probit models. *p* values were adjusted within comparison families by the Benjamini–Hochberg procedure; *q* < 0.05 denotes a model-based difference between tested concentration–mortality curves, not a confirmed storage-condition effect. The fresh-oil (time-zero) bioassays were retained as descriptive context but excluded from longitudinal inference because no fresh-oil sample was assayed concurrently at the subsequent 4-, 8-, and 12-month dates. Because storage containers were not replicated, these comparisons are exploratory and their *q* values must not be interpreted as replicated inference on the effects of storage temperature or light.

## 3. Results

### 3.1. PAEO Composition

The initial GC-MS analysis identified 11 constituents in *Piper aduncum* essential oil (PAEO), accounting for 98.92% of the total chromatographic area (Table 1). Phenylpropanoids represented 81.06% of the identified profile, whereas sesquiterpenoid compounds accounted for 16.66%. Aliphatic hydrocarbons represented the remaining 1.20%. Dillapiole was the predominant constituent (70.34%), followed by myristicin (9.57%), viridiflorol (4.68%), (E)-caryophyllene (3.82%), caryophyllene oxide (2.38%), and spathulenol (2.36%). Together, these six compounds represented 93.15% of the chromatographic area, with dillapiole and myristicin contributing 79.91%.

Within the sesquiterpenoid fraction, oxygenated compounds—viridiflorol, caryophyllene oxide, and spathulenol—accounted for 9.42% of the chromatographic area, whereas sesquiterpene hydrocarbons contributed 7.24%. The remaining five constituents were present at low relative abundances, each accounting for no more than 1.42%. Overall, the initial PAEO profile was characterized by a marked predominance of dillapiole and a smaller contribution from oxygenated and hydrocarbon sesquiterpenoids. Because chemical characterization was performed on a single initial sample, the reported values represent relative chromatographic peak areas rather than replicate-based estimates of chemical composition.

### 3.2. Toxicological Response After Four Months of Storage

After four months of storage, the binomial-probit models adequately described the concentration-mortality responses of all four species, with no significant lack of fit across the evaluated storage conditions (goodness-of-fit *p* = 0.053–0.976; Table 2). *Cryptolestes ferrugineus* was the most susceptible species, with LC_50_ values of 0.008–0.009 µL cm^−2^ and LC_95_ values of 0.015–0.018 µL cm^−2^. Its dose–response slopes were comparatively steep (5.98–6.53). Model-based comparisons showed differences among the tested samples in both storage experiments at four months (*q* < 0.001 for both), with higher LC_50_ values at 35 °C and under light exposure. Despite these differences in the fitted curves, LC_50_ estimates remained within a narrow range across the five storage conditions.

For *Oryzaephilus surinamensis*, the exploratory temperature and light comparisons each detected dose–response differences at four months (*q* < 0.001 for both). LC_50_ increased from 0.033 µL cm^−2^ at 5 °C to 0.040 µL cm^−2^ at 20 °C and 0.062 µL cm^−2^ at 35 °C. Light-exposed PAEO also had a higher LC_50_ (0.076 µL cm^−2^) than light-protected PAEO (0.041 µL cm^−2^). LC_95_ estimates were less precise than LC_50_ estimates.

*Sitophilus zeamais* also showed model-based differences across the temperature samples (q = 0.010) and between the light samples (*q* = 0.021). LC_50_ values were similar at 5 and 20 °C (0.276 and 0.269 µL cm^−2^) but increased to 0.388 µL cm^−2^ at 35 °C. Light-exposed PAEO likewise had a higher LC_50_ (0.376 µL cm^−2^) than light-protected PAEO (0.274 µL cm^−2^).

*Tribolium castaneum* was the least susceptible species at four months, with LC_50_ values of 1.522–1.899 µL cm^−2^ and LC_95_ values of 4.696–6.095 µL cm^−2^. The exploratory temperature contrast was not significant (*q* = 0.183), whereas the light contrast differed between the tested samples (*q* < 0.001). Across corresponding storage conditions, its LC_50_ estimates were approximately 200 times those of *C. ferrugineus*, highlighting the marked difference in susceptibility between the two species.

### 3.3. Toxicological Response After Eight Months of Storage

After eight months, the probit models continued to fit the concentration-mortality data well for all species and storage conditions (goodness-of-fit *p* = 0.494–0.929; Table 3). *Cryptolestes ferrugineus* remained the most susceptible species, with LC_50_ values of 0.008–0.010 µL cm^−2^ and LC_95_ values of 0.015–0.017 µL cm^−2^. Differences between fitted curves were detected among the temperature samples and between the two light-storage samples (*q* < 0.001 for both).

For *O. surinamensis*, the exploratory temperature (*q* = 0.001) and light (*q* = 0.008) contrasts remained significant between the tested samples. LC_50_ values were 0.059, 0.060, and 0.092 µL cm^−2^ at 5, 20, and 35 °C, respectively, and increased from 0.066 µL cm^−2^ under light protection to 0.096 µL cm^−2^ under light exposure.

For *S. zeamais*, exploratory contrasts identified differences in the tested samples in both experiments (temperature, *q* < 0.001; light, *q* = 0.003). LC_50_ rose from 0.348 µL cm^−2^ at 5 °C and 0.365 µL cm^−2^ at 20 °C to 0.579 µL cm^−2^ at 35 °C. Light-exposed PAEO had an LC_50_ of 0.529 µL cm^−2^, compared with 0.333 µL cm^−2^ for light-protected PAEO.

*T. castaneum* again had the highest lethal-concentration estimates. The exploratory temperature contrast was not significant at eight months (*q* = 0.282), with LC_50_ values between 1.666 and 1.836 µL cm^−2^. The exploratory light contrast remained significant between the two stored-oil samples (*q* < 0.001), increasing LC_50_ from 1.574 to 2.014 µL cm^−2^.

### 3.4. Toxicological Response After Twelve Months of Storage

At 12 months, *C. ferrugineus* remained highly susceptible to PAEO, with LC_50_ values of 0.004–0.005 µL cm^−2^ and LC_95_ values of 0.013–0.014 µL cm^−2^ (Table 4). The linear probit model showed extra-binomial variation and/or lack of fit under all five storage conditions for this species; Pearson-scaled standard errors and confidence intervals were therefore used. The exploratory temperature contrast remained significant across the tested samples (*q* = 0.031), whereas the light contrast did not (*q* = 0.949).

For *O. surinamensis*, the exploratory temperature contrast remained significant at 12 months (*q* < 0.001), but the light contrast did not (*q* = 0.690). The highest LC_50_ occurred at 35 °C (0.019 µL cm^−2^), compared with 0.012 µL cm^−2^ at 5 °C and 0.009 µL cm^−2^ at 20 °C. Light-protected and light-exposed storage produced nearly identical LC_50_ estimates (0.009 µL cm^−2^).

For *S. zeamais*, neither exploratory contrast reached significance at 12 months (temperature, *q* = 0.277; light, *q* = 0.525). LC_50_ values ranged from 0.146 to 0.202 µL cm^−2^ among temperatures and from 0.141 to 0.176 µL cm^−2^ between light conditions.

No significant within-time model-based contrast of temperature (*q* = 0.814) or light (*q* = 0.277) was detected for *T. castaneum* at 12 months. LC_50_ values were 1.836, 1.885, and 1.991 µL cm^−2^ at 5, 20, and 35 °C, respectively, and 1.846 and 2.038 µL cm^−2^ under light-protected and light-exposed storage.

### 3.5. Exploratory Longitudinal Comparisons Among Assayed Oil Samples

The longitudinal binomial-probit models detected variation among the three assessment occasions for all four species in both experiments after Benjamini–Hochberg correction (Table 5). This time-associated variation should not be interpreted as an isolated effect of storage duration, because insect cohorts and other date-related factors were not independently controlled. The association was pronounced for *C. ferrugineus*, *O. surinamensis*, and *S. zeamais* (all *q* < 0.001), whereas for *T. castaneum* it was smaller but met the nominal *q* threshold in the temperature (*q* = 0.025) and light (*q* = 0.047) experiments.

Within the two separately analyzed experiments, exploratory model comparisons identified differences in dose–response curves among the samples tested for *C. ferrugineus*, *O. surinamensis*, and *S. zeamais* in both the temperature and light series (all *q* ≤ 0.002). For *T. castaneum*, the overall light contrast met the nominal *q* threshold (*q* < 0.001), whereas the temperature contrast was not (*q* = 0.288). No exploratory time × condition comparison reached *q* < 0.05 after false-discovery-rate correction (*q* = 0.393–0.997). Temperature and light effects cannot be compared as though they were factors in a crossed design, and the results do not quantify their combined effect. As a single bottle was used for each storage condition, these tests compare the responses to the oil samples tested; they do not provide independent replication of the storage treatments.

Temporal trajectories differed among species rather than following a common decline in activity. In *C. ferrugineus*, LC_50_ values changed little from 4 to 8 months and then decreased at 12 months. *O. surinamensis* and *S. zeamais* generally showed higher LC_50_ values at 8 months followed by lower values at 12 months. Depending on storage condition, the 12-month LC_50_ represented approximately 12–35% of the four-month value for *O. surinamensis* and 47–60% for *S. zeamais*.

*T. castaneum* changed comparatively little over time: its 12-month LC_50_ estimates were generally 1.07–1.21 times the corresponding four-month values. Despite temporal variation, the relative susceptibility ranking was stable across conditions: *C. ferrugineus* was the most susceptible species, followed by *O. surinamensis*, *S. zeamais*, and *T. castaneum*.

**Figure 2:**
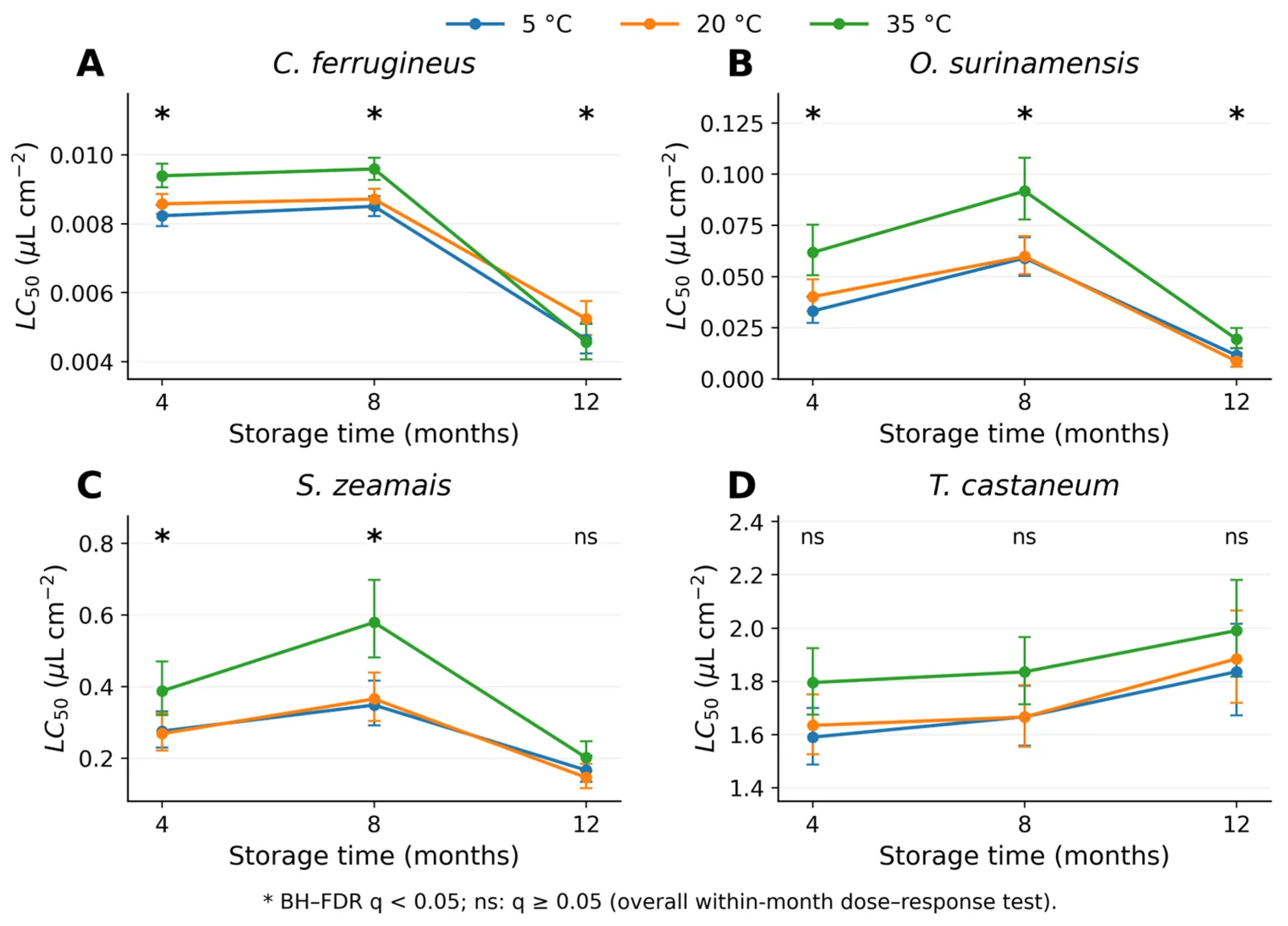
Temporal variation in LC_50_ estimates of *Piper aduncum* essential oil (PAEO) stored at 5, 20, and 35 °C against (**A**) *Cryptolestes ferrugineus*, (**B**) *Oryzaephilus surinamensis*, (**C**) *Sitophilus zeamais*, and (**D**) *Tribolium castaneum*. Error bars represent 95% confidence intervals. Symbols indicate exploratory overall within-month temperature comparisons (*, BH-adjusted *q* < 0.05; ns, not significant), not pairwise tests or independently replicated storage effects. See Table 6 for adjusted *q* values.

Within individual assessment times, the model-based temperature contrasts were most evident at 4 and 8 months. Storage at 35 °C generally produced higher LC_50_ values than storage at 5 or 20 °C for *C. ferrugineus*, *O. surinamensis*, and *S. zeamais* (Table 6; Figure 2). By 12 months, temperature-related differences had weakened, although *O. surinamensis* still showed its highest LC_50_ at 35 °C.

**Figure 3:**
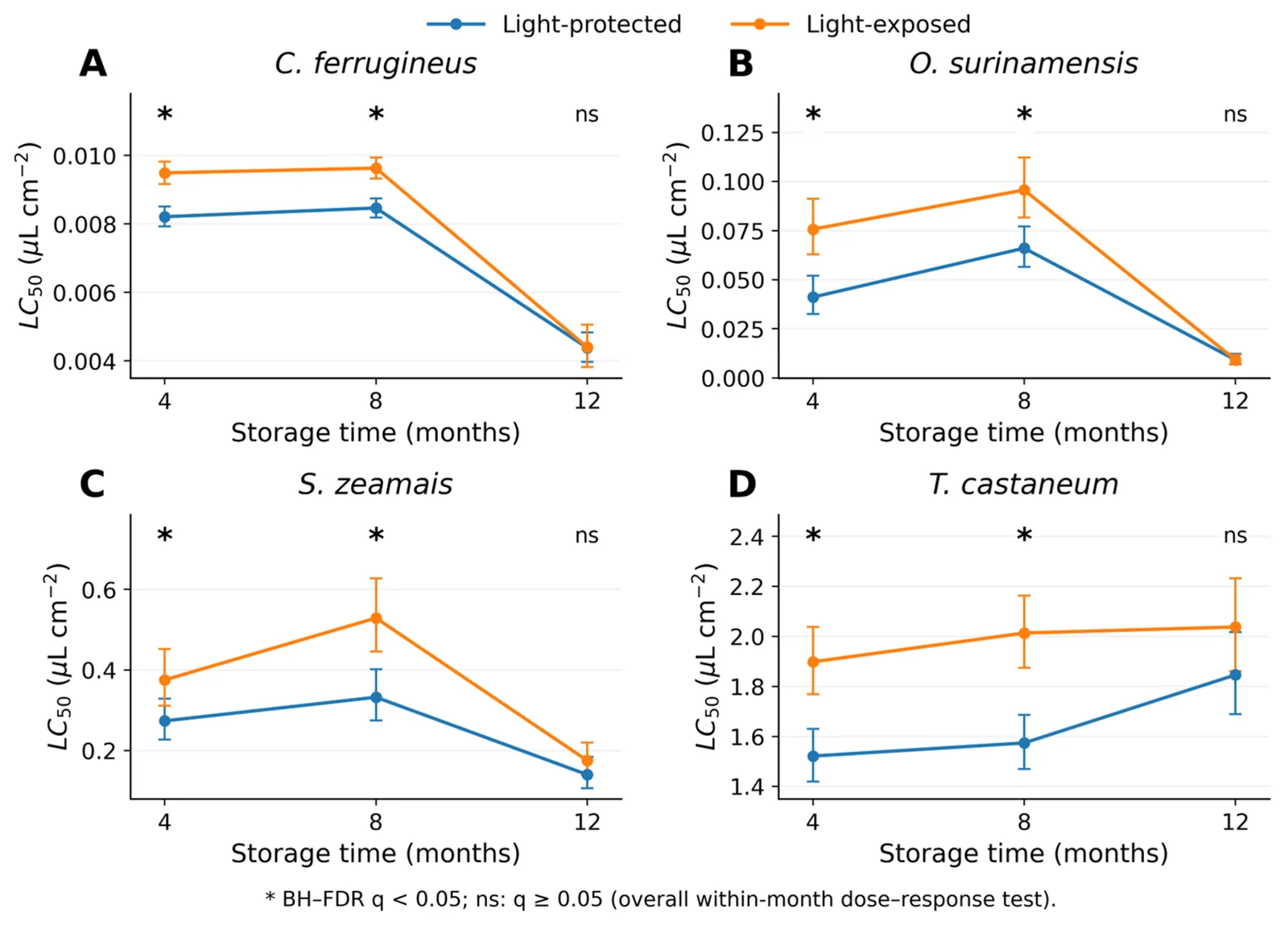
Temporal variation in LC_50_ estimates of *Piper aduncum* essential oil (PAEO) under light-protected and light-exposed storage against (**A**) *Cryptolestes ferrugineus*, (**B**) *Oryzaephilus surinamensis*, (**C**) *Sitophilus zeamais*, and (**D**) *Tribolium castaneum*. Error bars represent 95% confidence intervals. Symbols indicate exploratory within-month light-condition comparisons (*, BH-adjusted *q* < 0.05; ns, not significant), not independently replicated storage effects. See Table 6 for adjusted *q* values.

The separate light series showed a consistent descriptive contrast during the first eight months. For all four species, light-exposed PAEO had higher LC_50_ values than light-protected PAEO at both 4 and 8 months, whereas no exploratory light contrast reached *q* < 0.05 at 12 months (Table 6; Figure 3).

PAEO caused insect mortality in the laboratory bioassays throughout the 12-month observation period, with marked differences among insect species, assessment dates, and storage treatments. Temporal patterns were often non-monotonic; because fresh PAEO was not reassayed at each later date, these findings do not establish a progressive storage-aging trajectory.

## 4. Discussion

The initial GC-MS profile was dominated by phenylpropanoids and sesquiterpenoid constituents, with dillapiole accounting for 70.34% of the identified composition. Previous studies have documented substantial chemical variation in *Piper aduncum* oils, including profiles dominated by apiol [32], dillapiole-rich material from the Amazon region [33], and a dillapiole-rich population among chemically diverse populations from Central Brazil [34]. PAEO is active against several stored-product pests, including *Sitophilus zeamais*, *Sitophilus oryzae*, *Tenebrio molitor*, and *Cryptolestes ferrugineus* [9,10,13,35]. Essential-oil activity can reflect interactions among multiple constituents [36], and synergistic effects of dillapiole-rich PAEO have been reported with *Spodoptera frugiperda* [37,38] and *S. zeamais* [12]. Methylenedioxyphenyl-containing compounds, including dillapiole, can interfere with insect detoxification systems such as cytochrome P450 monooxygenases and esterases [39,40].

Apparent susceptibility differed among the four target species: *C. ferrugineus* consistently required the lowest concentrations, followed by *O. surinamensis* and *S. zeamais*, whereas *T. castaneum* required the highest. PAEO remained insecticidally active at every assessment. Nevertheless, the categorical assessment-time effects from the longitudinal models cannot be attributed solely to chemical aging, because assays conducted after 4, 8, and 12 months were performed on different dates and lacked concurrent fresh-oil controls.

For the individual bottles evaluated, the separate temperature experiment showed generally higher LC_50_ values following storage at 35 °C than at 5 or 20 °C for *C. ferrugineus*, *O. surinamensis*, and *S. zeamais* at 4 and 8 months. The contrast was weaker at 12 months, although *O. surinamensis* still showed its highest LC_50_ at 35 °C. These comparisons were made only among light-protected bottles. They should not be generalized to temperature effects under illumination or to a joint temperature–light interaction.

Volatilization, oxidation, and other chemical reactions have been documented for constituents of essential oils during storage [20,23,41,42]. Studies on storage temperature and heating have also documented compositional changes in essential oils [4,43,44]. Such processes are plausible explanations for some differences in insecticidal activity, but none was measured in the present experiment. Because only an initial GC-MS composition profile is available, there is no direct evidence that the observed bioassay differences resulted from changes in dillapiole or other constituents. Mechanistic explanations therefore remain hypotheses requiring longitudinal chemical analysis.

In the light experiment conducted at 20 °C, light-exposed PAEO had higher LC_50_ values than light-protected PAEO for all four species at 4 and 8 months; the exploratory within-time light contrast did not reach *q* < 0.05 at 12 months (Table 6; Figure 3). Light-associated oxidation is a possible mechanism based on previous studies [21,23,25,26], but this study did not measure photo-oxidation products or repeated chemical composition. Because the protected and illuminated samples also differed in bottle transparency and covering, this is a comparison of storage arrangements at 20 °C rather than an isolated irradiance effect.

The temporal trajectories did not follow a simple pattern of progressive deterioration. LC_50_ changed little between 4 and 8 months for *C. ferrugineus* and then fell at 12 months. In *O. surinamensis* and *S. zeamais*, LC_50_ generally rose at 8 months and then declined at 12 months, whereas *T. castaneum* changed comparatively little. The exploratory time × condition comparisons did not reach the nominal significance threshold after false-discovery-rate correction; this finding should not be interpreted as evidence for constant storage-treatment effects.

The pronounced non-monotonic differences among assay dates require particular caution. Unlike a design in which fresh and stored samples are tested concurrently at each date, the present study compared stored-oil bioassays performed at separate occasions. Changes in insect cohort, rearing batch, colony age structure, physiological condition, or subtle assay-environment differences could have contributed to the estimated time effect. Seasonal changes in laboratory colonies cannot be excluded either. Thus, the categorical time term describes the combined association of storage duration and assessment-specific biological or procedural variation, rather than an unconfounded storage-aging effect. Repeated opening of the same bottle to withdraw samples may also have changed air exposure and the proportion of volatile constituents, but these possible effects were not measured. Time-zero assays were therefore descriptive only. A repeated fresh-oil control at every assay date would be required for stronger causal attribution.

LC_95_ was less robust than LC_50_ because high-mortality quantiles frequently lay beyond the experimental concentration range. A direct comparison with the original per-assay maximum doses showed that 55 of the 60 LC_95_ point estimates in Table 2, Table 3 and Table 4 exceeded the highest tested concentration in their respective species × condition × time cells. They are consequently model-based extrapolations, and their confidence intervals—often wide—should be interpreted cautiously. At 12 months, *C. ferrugineus* also showed extra-binomial variability and lack of fit in the simple linear probit model, for which Pearson-scaled uncertainty estimates were used. LC_50_ is therefore emphasized as the more reliable comparative endpoint.

Earlier research reported maintenance of PAEO insecticidal activity and a high dillapiole proportion during storage [30], while changes in the relative abundance of constituents under different storage conditions have also been described [29]. These studies support the need to investigate chemical and toxicological stability together; they do not establish a chemical mechanism for the changes observed here. No intermediate or final GC-MS profiles were available for the present experiment, so oxidation, loss of volatiles, and degradation cannot be confirmed.

Limitations. Bioassays were performed under controlled laboratory conditions (25 ± 5 °C; 70 ± 5% relative humidity) using filter-paper contact exposure; the applied concentration ranges and exposure route need not reflect grain-bin applications or heterogeneous field populations, including populations with different resistance phenotypes. At 12 months, *T. castaneum* had two dish replicates per concentration because colony numbers were insufficient for a third, reducing precision relative to the other assays. Each storage condition was represented by one PAEO bottle, whereas the Petri dishes provided replication only at the bioassay level. Accordingly, the model-based contrasts cannot be generalized as independently replicated storage-treatment effects. The same bottle was opened at each four-month sampling, so additional air exposure and volatile losses may have contributed to temporal differences; these effects were not quantified. In addition, the assay-date effect is confounded with possible insect-batch differences, most LC_95_ values require extrapolation, and GC-MS was performed only initially. These limitations restrict causal conclusions about storage chemistry and generalization to practical storage facilities.

Taken together, the data document persisting laboratory insecticidal activity at the investigated assessment dates. In the temperature-only experiment, avoiding 35 °C generally coincided with lower LC_50_ at 4 and 8 months for three species; in the separate light experiment held at 20 °C, protected samples generally had lower LC_50_ across the same dates. Neither comparison establishes combined protection from heat and light, and the observed model-based differences require confirmation using independently replicated storage containers, concurrent fresh-oil assays, and longitudinal chemistry.

## 5. Conclusions

*Piper aduncum* essential oil remained insecticidally active in laboratory contact bioassays after 4, 8, and 12 months under the conditions studied. Within the light-protected temperature experiment, storage at 35 °C was generally associated with higher LC_50_ estimates during the first eight months for three species. In a separate experiment conducted at 20 °C, the light-exposed sample showed higher LC_50_ estimates at 4 and 8 months for all four species. Exploratory model-based *q* values describe the assayed samples, not confirmed causal storage-treatment effects. Differences among assay dates were not consistently progressive and may also reflect insect-cohort variation and repeated opening of the bottles; no fresh-oil control was tested alongside the stored oil. The findings concern measured toxicological activity, not demonstrated chemical stability or validated commercial shelf life. Further studies should incorporate independent storage-bottle replication, add parallel fresh-oil controls, and repeat GC-MS analyses over time.

## Figures and Tables

**Table 1:** Chemical composition and relative abundance of compounds identified by gas chromatography-mass spectrometry (GC-MS) in the PAEO used in the bioassays.

Components	Class	LRI ^a^	KI ^a^	LRI ^b^	KI ^b^	Relative Abundance (% Area)
α-Copaene	Sesquiterpenoid	1383	1386	1374	1376	0.96
(E)-Caryophyllene	Sesquiterpenoid	1429	1433	1417	1419	3.82
α-Humulene	Sesquiterpenoid	1464	1470	1452	1454	1.04
Germacrene-D	Sesquiterpenoid	1492	1499	1484	1485	1.42
Pentadecane	Aliphatic hydrocarbon	1504	1511	1500	1500	1.20
Myristicin	Phenylpropanoid	1534	1539	1517	1518	9.57
(+)-spathulenol ^c^	Sesquiterpenoid	1591	1592	–	–	2.36
Caryophyllene oxide	Sesquiterpenoid	1595	1596	1582	1583	2.38
Viridiflorol	Sesquiterpenoid	1605	1606	1592	1592	4.68
Dillapiole	Phenylpropanoid	1650	1653	1620	1620	70.34
Apiol	Phenylpropanoid	1698	1703	1677	1678	1.15
Total (identified)	–	–	–	–	–	98.92

Notes: LRI, linear retention index; KI, Kovats index. ^a^ Experimental retention indices calculated using an n-alkane series (C10–C40). ^b^ Reference retention indices reported in the literature [31]. ^c^ Compound tentatively identified by comparison with the NIST mass spectral library.

**Table 2:** Dose–response estimates for *Piper aduncum* essential oil (PAEO) after 4 months of storage under different temperature and light conditions against adults of four stored-product insect pests.

Species	Experiment	Storage Condition	Slope (±SE)	LC_50_ (95% CI) (µL cm^−2^)	LC_95_ (95% CI) (µL cm^−2^)	Pearson Dispersion	GOF *p*
*C. ferrugineus*	Temperature	5 °C	5.98 ± 0.39	0.008 (0.008–0.009)	0.015 (0.014–0.017)	0.95	0.509
	Temperature	20 °C	6.53 ± 0.40	0.009 (0.008–0.009)	0.015 (0.014–0.017)	0.84	0.646
	Temperature	35 °C	6.04 ± 0.42	0.009 (0.009–0.010)	0.018 (0.016–0.019)	1.09	0.354
	Light	Light-protected	6.25 ± 0.39	0.008 (0.008–0.009)	0.015 (0.014–0.016)	0.82	0.667
	Light	Light-exposed	6.51 ± 0.44	0.009 (0.009–0.010)	0.017 (0.016–0.018)	1.12	0.330
*O. surinamensis*	Temperature	5 °C	1.12 ± 0.07	0.033 (0.027–0.040)	0.969 (0.629–1.492)	0.82	0.667
	Temperature	20 °C	1.12 ± 0.07	0.040 (0.033–0.049)	1.193 (0.765–1.859)	0.81	0.675
	Temperature	35 °C	1.06 ± 0.07	0.062 (0.051–0.075)	2.236 (1.343–3.722)	0.90	0.566
	Light	Light-protected †	1.17 ± 0.09	0.041 (0.033–0.052)	1.034 (0.609–1.757)	1.63	0.053
	Light	Light-exposed	1.16 ± 0.07	0.076 (0.063–0.091)	1.996 (1.261–3.159)	0.43	0.976
*S. zeamais*	Temperature	5 °C	1.24 ± 0.09	0.276 (0.229–0.331)	5.876 (3.992–8.650)	0.56	0.917
	Temperature	20 °C	1.19 ± 0.09	0.269 (0.222–0.325)	6.527 (4.324–9.854)	0.90	0.569
	Temperature	35 °C	1.09 ± 0.08	0.388 (0.320–0.469)	12.546 (7.548–20.856)	0.62	0.872
	Light	Light-protected	1.24 ± 0.09	0.274 (0.228–0.329)	5.802 (3.948–8.527)	0.77	0.723
	Light	Light-exposed	1.13 ± 0.08	0.376 (0.312–0.452)	10.587 (6.608–16.962)	0.60	0.887
*T. castaneum*	Temperature	5 °C	3.50 ± 0.19	1.591 (1.488–1.700)	4.696 (4.101–5.376)	0.64	0.855
	Temperature	20 °C	3.33 ± 0.19	1.635 (1.526–1.751)	5.102 (4.413–5.898)	0.81	0.678
	Temperature	35 °C	3.31 ± 0.19	1.796 (1.675–1.925)	5.629 (4.838–6.549)	0.76	0.730
	Light	Light-protected	3.30 ± 0.18	1.522 (1.420–1.631)	4.802 (4.161–5.541)	0.79	0.698
	Light	Light-exposed	3.25 ± 0.19	1.899 (1.768–2.039)	6.095 (5.196–7.149)	0.68	0.816

Notes: LC_50_ and LC_95_: concentrations estimated to cause 50% and 95% mortality, respectively; CI: 95% confidence interval; SE: standard error; GOF *p*: Pearson goodness-of-fit *p* value. Each concentration was represented by three independent Petri-dish bioassay replicates of 50 adults. † Model flagged for extra-binomial variation and/or significant lack of fit; Pearson-scaled standard errors and confidence intervals are reported. LC_95_ estimates above the maximum tested concentration are model-based extrapolations and should be interpreted cautiously.

**Table 3:** Dose–response estimates for *Piper aduncum* essential oil (PAEO) after 8 months of storage under different temperature and light conditions against adults of four stored-product insect pests.

Species	Experiment	Storage Condition	Slope (±SE)	LC_50_ (95% CI) (µL cm^−2^)	LC_95_ (95% CI) (µL cm^−2^)	Pearson Dispersion	GOF *p*
*C. ferrugineus*	Temperature	5 °C	6.37 ± 0.40	0.009 (0.008–0.009)	0.015 (0.014–0.017)	0.57	0.911
	Temperature	20 °C	6.41 ± 0.40	0.009 (0.008–0.009)	0.016 (0.015–0.017)	0.73	0.764
	Temperature	35 °C	6.36 ± 0.41	0.010 (0.009–0.010)	0.017 (0.016–0.019)	0.81	0.676
	Light	Light-protected	6.63 ± 0.41	0.008 (0.008–0.009)	0.015 (0.014–0.016)	0.74	0.751
	Light	Light-exposed	6.77 ± 0.43	0.010 (0.009–0.010)	0.017 (0.016–0.018)	0.95	0.510
*O. surinamensis*	Temperature	5 °C	1.42 ± 0.08	0.059 (0.050–0.069)	0.849 (0.604–1.192)	0.96	0.494
	Temperature	20 °C	1.47 ± 0.08	0.060 (0.051–0.070)	0.781 (0.564–1.082)	0.83	0.654
	Temperature	35 °C	1.41 ± 0.08	0.092 (0.078–0.108)	1.358 (0.941–1.959)	0.82	0.660
	Light	Light-protected †	1.48 ± 0.08	0.066 (0.057–0.077)	0.850 (0.612–1.179)	1.00	0.449
	Light	Light-exposed	1.46 ± 0.08	0.096 (0.082–0.112)	1.276 (0.897–1.815)	0.86	0.611
*S. zeamais*	Temperature	5 °C	1.21 ± 0.09	0.348 (0.292–0.416)	7.955 (5.243–12.070)	0.78	0.705
	Temperature	20 °C	1.15 ± 0.08	0.365 (0.304–0.439)	9.765 (6.187–15.412)	0.65	0.843
	Temperature	35 °C	1.08 ± 0.08	0.579 (0.481–0.697)	19.105 (10.942–33.358)	0.63	0.863
	Light	Light-protected	1.13 ± 0.08	0.333 (0.275–0.402)	9.471 (5.960–15.049)	0.54	0.929
	Light	Light-exposed	1.20 ± 0.09	0.529 (0.446–0.627)	12.330 (7.798–19.495)	0.83	0.648
*T. castaneum*	Temperature	5 °C	3.46 ± 0.19	1.667 (1.559–1.783)	4.980 (4.332–5.724)	0.70	0.793
	Temperature	20 °C	3.29 ± 0.18	1.666 (1.554–1.786)	5.270 (4.544–6.112)	0.73	0.770
	Temperature	35 °C	3.37 ± 0.19	1.836 (1.714–1.967)	5.642 (4.858–6.553)	0.83	0.648
	Light	Light-protected	3.32 ± 0.18	1.574 (1.469–1.686)	4.921 (4.263–5.679)	0.82	0.662
	Light	Light-exposed	3.29 ± 0.19	2.014 (1.875–2.163)	6.378 (5.423–7.501)	0.63	0.861

Notes: LC_50_ and LC_95_: concentrations estimated to cause 50% and 95% mortality, respectively; CI: 95% confidence interval; SE: standard error; GOF *p*: Pearson goodness-of-fit *p* value. Each concentration was represented by three independent Petri-dish bioassay replicates of 50 adults. † Model flagged for extra-binomial variation and/or significant lack of fit; Pearson-scaled standard errors and confidence intervals are reported. LC_95_ estimates above the maximum tested concentration are model-based extrapolations and should be interpreted cautiously.

**Table 4:** Dose–response estimates for *Piper aduncum* essential oil (PAEO) after 12 months of storage under different temperature and light conditions against adults of four stored-product insect pests.

Species	Experiment	Storage Condition	Slope (±SE)	LC_50_ (95% CI) (µL cm^−2^)	LC_95_ (95% CI) (µL cm^−2^)	Pearson Dispersion	GOF *p*
*C. ferrugineus*	Temperature	5 °C	3.62 ± 0.31	0.005 (0.004–0.005)	0.013 (0.011–0.016)	1.98	0.011
	Temperature	20 °C	3.94 ± 0.39	0.005 (0.005–0.006)	0.014 (0.011–0.017)	2.51	<0.001
	Temperature	35 °C	3.57 ± 0.38	0.005 (0.004–0.005)	0.013 (0.011–0.016)	3.10	<0.001
	Light	Light-protected	3.59 ± 0.32	0.004 (0.004–0.005)	0.013 (0.011–0.015)	2.17	0.004
	Light	Light-exposed	3.51 ± 0.44	0.004 (0.004–0.005)	0.013 (0.010–0.017)	4.28	<0.001
*O. surinamensis*	Temperature	5 °C	1.29 ± 0.11	0.011 (0.009–0.015)	0.219 (0.143–0.334)	1.49	0.093
	Temperature	20 °C	1.03 ± 0.11	0.009 (0.006–0.013)	0.339 (0.184–0.622)	1.94	0.013
	Temperature	35 °C	1.09 ± 0.09	0.019 (0.015–0.025)	0.628 (0.382–1.033)	1.34	0.161
	Light	Light-protected †	1.18 ± 0.10	0.009 (0.007–0.012)	0.231 (0.146–0.364)	1.45	0.107
	Light	Light-exposed	1.09 ± 0.08	0.009 (0.007–0.012)	0.302 (0.200–0.457)	0.95	0.511
*S. zeamais*	Temperature	5 °C	1.21 ± 0.09	0.167 (0.134–0.207)	3.784 (2.608–5.491)	0.66	0.840
	Temperature	20 °C	1.21 ± 0.09	0.146 (0.116–0.184)	3.363 (2.326–4.861)	0.91	0.555
	Temperature	35 °C	1.23 ± 0.09	0.202 (0.165–0.247)	4.362 (3.007–6.330)	0.80	0.691
	Light	Light-protected	1.03 ± 0.09	0.141 (0.107–0.185)	5.586 (3.480–8.967)	0.71	0.789
	Light	Light-exposed	1.14 ± 0.09	0.176 (0.140–0.221)	4.919 (3.250–7.446)	0.94	0.525
*T. castaneum*	Temperature	5 °C	3.03 ± 0.22	1.836 (1.673–2.016)	6.419 (5.336–7.722)	1.04	0.409
	Temperature	20 °C	3.07 ± 0.22	1.885 (1.719–2.067)	6.465 (5.380–7.769)	1.04	0.402
	Temperature	35 °C	2.99 ± 0.22	1.991 (1.818–2.180)	7.064 (5.839–8.547)	0.79	0.636
	Light	Light-protected	3.17 ± 0.22	1.846 (1.690–2.017)	6.098 (5.137–7.238)	0.93	0.502
	Light	Light-exposed	2.98 ± 0.22	2.038 (1.861–2.232)	7.264 (5.986–8.814)	0.72	0.706

Notes: LC_50_ and LC_95_: concentrations estimated to cause 50% and 95% mortality, respectively; CI: 95% confidence interval; SE: standard error; GOF *p*: Pearson goodness-of-fit *p* value. Each concentration was represented by three independent Petri-dish bioassay replicates of 50 adults, except for *T. castaneum*, for which two replicates per concentration were available. † Model flagged for extra-binomial variation and/or significant lack of fit; Pearson-scaled standard errors and confidence intervals are reported. LC_95_ estimates above the maximum tested concentration are model-based extrapolations and should be interpreted cautiously.

**Table 5:** Exploratory comparisons of assessment time, storage condition, and their interaction in PAEO concentration–mortality responses. Temperature and light experiments were analyzed separately.

Species	Storage Experiment	Time	Storage Condition	Time × Condition
*C. ferrugineus*	Temperature	F(12, 144) = 63.19; *p* < 0.001; *q* < 0.001	F(12, 144) = 4.16; *p* < 0.001; *q* < 0.001	F(8, 144) = 1.72; *p* = 0.098; *q* = 0.393
	Light	F(8, 96) = 67.68; *p* < 0.001; *q* < 0.001	F(6, 96) = 6.61; *p* < 0.001; *q* < 0.001	F(4, 96) = 2.07; *p* = 0.090; *q* = 0.393
*O. surinamensis*	Temperature	F(12, 144) = 40.96; *p* < 0.001; *q* < 0.001	F(12, 144) = 5.91; *p* < 0.001; *q* < 0.001	F(8, 144) = 0.83; *p* = 0.578; *q* = 0.771
	Light	F(8, 96) = 70.60; *p* < 0.001; *q* < 0.001	F(6, 96) = 5.19; *p* < 0.001; *q* < 0.001	F(4, 96) = 1.61; *p* = 0.177; *q* = 0.472
*S. zeamais*	Temperature	F(12, 144) = 13.90; *p* < 0.001; *q* < 0.001	F(12, 144) = 3.46; *p* < 0.001; *q* < 0.001	F(8, 144) = 0.51; *p* = 0.850; *q* = 0.972
	Light	F(8, 96) = 13.85; *p* < 0.001; *q* < 0.001	F(6, 96) = 3.87; *p* = 0.002; *q* = 0.002	F(4, 96) = 1.35; *p* = 0.257; *q* = 0.515
*T. castaneum*	Temperature	F(12, 126) = 2.09; *p* = 0.022; *q* = 0.025	F(12, 126) = 1.20; *p* = 0.288; *q* = 0.288	F(8, 126) = 0.15; *p* = 0.997; *q* = 0.997
	Light	F(8, 84) = 2.07; *p* = 0.047; *q* = 0.047	F(6, 84) = 7.69; *p* < 0.001; *q* < 0.001	F(4, 84) = 1.08; *p* = 0.370; *q* = 0.592

Notes: *p* values are dispersion-adjusted model-comparison tests from binomial-probit models; *q* values are Benjamini–Hochberg-adjusted (*q* < 0.05). Comparisons are exploratory, as each storage condition had one oil bottle and assessment time may be confounded with assay date. Temperature (5, 20, and 35 °C) and light (at 20 °C only) were evaluated separately; their interaction was not tested.

**Table 6:** Exploratory within-time comparisons of concentration–mortality responses among individual PAEO storage samples for each insect species.

Species	Month	Experiment	LRT	df	*p*	BH-FDR *q*
*C. ferrugineus*	4	Temperature	30.38	4	<0.001	<0.001
		Light	35.71	2	<0.001	<0.001
	8	Temperature	27.78	4	<0.001	<0.001
		Light	30.74	2	<0.001	<0.001
	12	Temperature	11.61	4	0.021	0.031
		Light	0.1	2	0.949	0.949
*O. surinamensis*	4	Temperature	21.84	4	<0.001	<0.001
		Light	21.18	2	<0.001	<0.001
	8	Temperature	19.3	4	<0.001	0.001
		Light	10.78	2	0.005	0.008
	12	Temperature	37.03	4	<0.001	<0.001
		Light	0.92	2	0.633	0.690
*S. zeamais*	4	Temperature	14.43	4	0.006	0.010
		Light	8.65	2	0.013	0.021
	8	Temperature	21.38	4	<0.001	<0.001
		Light	13.03	2	0.001	0.003
	12	Temperature	5.76	4	0.218	0.277
		Light	1.56	2	0.459	0.525
*T. castaneum*	4	Temperature	7.12	4	0.130	0.183
		Light	19.18	2	<0.001	<0.001
	8	Temperature	5.56	4	0.235	0.282
		Light	23.9	2	<0.001	<0.001
	12	Temperature	1.76	4	0.780	0.814
		Light	3.03	2	0.220	0.277

Notes: LRT: likelihood-ratio test; df: degrees of freedom; *q*: Benjamini–Hochberg false-discovery-rate adjusted *p* value. *q* < 0.05 was considered statistically significant. These *q* values summarize differences between curves fitted to the bioassays of the particular storage bottles; they are not tests of independently replicated storage-treatment effects.

## Data Availability

The datasets generated and/or analyzed during the current study are available from the corresponding author upon reasonable request.

## Abbreviations

The following abbreviations are used in this manuscript:

PAEO	*Piper aduncum* L. essential oil
GC-MS	Gas chromatography-mass spectrometry
LC_50_	Lethal concentration estimated to cause 50% mortality
LC_95_	Lethal concentration estimated to cause 95% mortality
CI	Confidence interval
LRI	Linear retention index
KI	Kovats index
BH-FDR	Benjamini–Hochberg false-discovery-rate adjustment
